# Direct Intrahepatic Portocaval Shunt for Sinusoidal Obstruction Syndrome Associated with Hepatotoxicity of Pyrrolizidine Alkaloids

**DOI:** 10.1155/2018/9804582

**Published:** 2018-06-13

**Authors:** Shihua Luo, Jianguo Chu, He Huang, Kechun Yao

**Affiliations:** ^1^Department of Gastroenterology, The First Affiliated Hospital, Wenzhou Medical University, Wenzhou 325000, Zhejiang Province, China; ^2^Department of Radiology, Air Force General Hospital of PLA, Haidian District, Beijing, 100142, China; ^3^Department of Ultrasound, Air Force General Hospital of PLA, Haidian District, Beijing, 100142, China

## Abstract

We retrospectively identified 89 consecutive patients from January 2004 to January 2012 to investigate efficacy of direct intrahepatic portocaval shunt (DIPS) combined with inferior vena cava (IVC) stenting for sinusoidal obstruction syndrome (SOS) associated with hepatotoxicity of pyrrolizidine alkaloids. Indications for treatment were variceal hemorrhage and/or refractory ascites. Patients were treated with DIPS plus IVC stenting (group A, n=68) or DIPS alone (group B, n=21). A technical success rate of 100% was obtained in all 89 patients, and there were no early procedure-related adverse events or 30-day mortality. Mean portosystemic gradient decreased in both groups. Changes in aspartate and alanine aminotransferases and total bilirubin did not differ between the groups. Ascites disappeared in group A but was not obvious in group B until IVC stenting. During follow-up, recurrent bleeding and ascites and incidence of hepatic encephalopathy did not differ between the groups. The 1-, 3-, and 5-year survival rate was 98, 89.59, and 80%, respectively. Satisfactory clinical results were obtained for combined DIPS and IVC stenting for SOS associated with pyrrolizidine-alkaloid-related decompensated cirrhosis.

## 1. Introduction

Sinusoidal obstruction syndrome (SOS) is a rare vascular disease of the liver, which can lead to lethal liver failure and portal-hypertension-related complications [1]. The clinical syndrome is characterized [2] by hepatomegaly, ascites, weight gain, increased aminotransferase levels, and jaundice due to sinusoidal congestion, which can be caused by alkaloid ingestion [3], hematopoietic stem cell transplantation (HSCT) [4], bone marrow transplantation, and radiation-induced liver disease or chemotherapy, and it is also seen after solid organ transplantation [5]. The most common cause of SOS in China is herbal medicines containing pyrrolizidine alkaloids, mostly Tusanqi (*Gynura segetum*) [6].

Transjugular intrahepatic portosystemic shunt (TIPS) was introduced as an alternative treatment for complications of portal hypertension [7]. TIPS has been progressively recognized as an effective therapeutic option in a growing number of clinical situations. Several case reports show the beneficial effects of TIPS in SOS [8]. It improved ascites and lowered the levels of aspartate aminotransferase (AST) and alanine aminotransferase (ALT), but not serum total bilirubin (TB) levels. Most of the patients died despite the creation of TIPS [9]. Although TIPS in SOS does reduce ascites, there is no survival benefit; therefore, SOS is not considered an appropriate indication for TIPS [10].

Direct intrahepatic portocaval shunt (DIPS) involves creation of a side-to-side shunt between the portal vein and inferior vena cava (IVC) via the caudate lobe of the liver [11]. A major advantage of DIPS is that it does not involve the hepatic vein; thus, it is especially useful in hepatic vein obstruction. However, patients who have SOS with hepatomegaly, or narrowed or obstructed IVC, may have undergone IVC stenting to recanalize the IVC blood flow.

In this study, we retrospectively evaluated 89 patients with SOS associated with hepatotoxicity of pyrrolizidine-alkaloid-related decompensated cirrhosis who underwent IVC stenting combined with DIPS between January 2004 and January 2012. Levels of AST, ALT, and TB, clinical outcomes, hepatic encephalopathy (HE), and mortality were compared between patients treated with combined DIPS and IVC stenting or DIPS alone.

## 2. Materials and Methods

### 2.1. Patient Information

Between January 2004 and January 2012, there were 127 patients with suspected SOS, and 38 patients were excluded due to lack of confirmation of SOS. We retrospectively identified 89 consecutive patients with proven SOS associated with hepatotoxicity of pyrrolizidine-alkaloid-related decompensated cirrhosis. Indications for stent graft shunt were variceal hemorrhage (n=35), refractory ascites (n=79), and both (n=27). The patients were divided into group A (DIPS plus IVC stenting, n=68) and group B (DIPS alone, n=21). We compared the clinical efficacy of the two treatment methods. The Ethics Committee approved the study protocol and all of the patients provided written informed consent. All procedures were conducted according to the guidelines approved by the Ethics Committee. We reviewed the patients' medical records and medical images to gather information regarding the underlying etiology, clinical presentation, age, sex, and severity of cirrhosis (Table 1).

### 2.2. Diagnosis of SOS

All cases had clear botanical hepatotoxicity caused by pyrrolizidine alkaloids, such as* Gynura segetum*, mushroom tea, and traditional remedies. Color Doppler ultrasound (US) was used for first-line diagnosis of SOS, and enhanced computed tomography (CT) and/or magnetic resonance imaging (MRI) were performed in all patients to confirm the US findings. SOS was diagnosed clinically referring to the Seattle or Baltimore criteria [12], the occurrence of two or more of the following events before day 21 after hemopoietic progenitor cell administration: hyperbilirubinemia (>34.2 *μ*mol/L or >2 mg/dL), ascites or sudden weight gain (>5% of baseline body weight), and painful hepatomegaly. No other explanation for these signs and symptoms (septicemia, cyclosporin toxicity, heart failure, hepatitis, etc.) could be present at the time of diagnosis.

Three cases underwent transjugular liver biopsy and 86 underwent percutaneous transhepatic biopsy to confirm the diagnosis of SOS. Diagnosis of liver cirrhosis depended upon assessing the history of liver disease, liver function, and liver imaging findings. The decompensated stage was defined by the presence of ascites, variceal bleeding, jaundice, or encephalopathy.

The exclusion criteria were as follows: patients with obscure botanical hepatotoxicity of pyrrolizidine alkaloids, lack of confirmation of SOS, severe right-sided heart failure, severe liver failure (bilirubin >4 mg/dL), polycystic liver disease, dilated biliary ducts, age >75 years, bilirubin level >5 mg/dL, creatinine level >3 mg/dL, Child–Pugh score >11, and diagnosis of hepatic carcinoma, sepsis, or spontaneous bacterial peritonitis.

### 2.3. IVC Stenting Procedure

Standard local anesthesia was used. Initially, patients were assessed for stenosis or occlusion of the hepatic segment of the IVC, which was confirmed by IVC angiography before the DIPS procedure. The criteria for IVC stent placement were severe stenosis and occlusion of the hepatic segment of the IVC. Balloon dilatation was done first, followed by stent placement (type Z, 25/75 mm, JRZ25-75; AT&M Biomaterials, Beijing, China), avoiding blood flow obstruction due to severe stenosis or occlusion of the IVC. DIPS was performed after 1 week.

### 2.4. DIPS Procedure

Standard local anesthesia was used. All patients were evaluated and followed up by the same medical team using a prospective protocol diagnostic work-up and surveillance strategy. Before DIPS, medical history was taken, and, after DIPS, the patients were followed up according to the same protocol.

DIPS was performed through a transjugular approach, as described previously [11]. After superior mesenteric artery angiography was performed, the outflow of the right hepatic vein was reached using a TIPS set (RUPS-100; Cook, Bloomington, IL, USA). The IVC wall was punctured with the RUPS-100 needle at the level of the hepatic segment from the junction of the hepatic vein and IVC to the left or right branch of the portal vein, under the guidance of digital subtraction angiography, in both the posterior anterior and lateral positions. When the branch of the portal vein was punctured and confirmed by portography, a 7–8-mm balloon (Cook) dilated the hepatic tract and a 7–8-mm expanded polytetrafluoroethylene stent graft (BARD; Fluency, Voisins le Bretonneux, France) were used for DIPS creation. Portosystemic gradient (PSG) and IVC pressure were measured before and after DIPS.

The entire length of the intrahepatic tract should be covered by the stent graft. The initial stent position in the distal inflow was to the main portal vein or the left branch of the portal vein, and the proximal outflow end was to the junction of the hepatic vein and IVC. If the IVC stenosis or occlusion had been recanalized by bare stent placement, the RUPS-100 needle was used to puncture the IVC stent at a hepatic segment level.

The shunts were dilated to their full nominal diameter to reach a target PSG of <12 mm Hg. Notable variceal collateral vessels observed during DIPS were embolized with coils (Cook), as long as the catheter could be inserted into the vessel. Covered stents (Viatorr; W.L. Gore & Associates, Flagstaff, AZ, USA) were not used because they are not approved by the China State Food and Drug Administration. Subsequent direct portography was performed to evaluate whether the portal venous system was completely patent. After DIPS, intravenous heparin (4000 U/day; Chase Sun Pharma, Tianjin, China) was given for 3 days and then oral warfarin (2.5 mg/day; Orion Pharma, Orionintie, Finland) was prescribed at doses to achieve an international normalized ratio of up 2.0.

### 2.5. Follow-Up

Baseline duplex US was performed on the day after DIPS. After the shunt procedure, patients were placed in an identical routine 5-year follow-up protocol. They were seen as outpatients 1 month after the procedure and then every 3 months or whenever needed. Each consultation included a clinical examination, blood chemistry, and an assessment of HE. Shunt occlusion needed for shunt revision during shunt venography or significant recurrent symptoms acted as the endpoint for loss of primary unassisted patency. Direct portography was performed in patients with recurrent symptoms of suspected shunt dysfunction. DIPS revision was performed when hemodynamically significant shunt stenosis (>50%) was present, when there was recurrent variceal bleeding, recurrent or gradually worsening ascites, or PSG was ≥15 mmHg, unless grade III/IV hepatic encephalopathy(HE) (West Haven Criteria) was present. Patients lost to follow-up were censored at the time of the last known imaging of the shunt (duplex US or shunt venography).

### 2.6. Statistical Analysis

Continuous variables were summarized as mean ± SD and compared using the independent sample *t*-test or one-way analysis of variance; categorical variables were expressed as frequencies and compared using *χ*^2^ tests. Differences were considered significant at* P*<0.05. The statistical analyses were performed with SPSS version 20.0 (Chicago, IL, USA).

## 3. Results

In both groups, mean PSG and levels of AST, ALT, and TB were decreased. In group A, ascites disappeared in the first week without paracentesis; the DIPS stent graft and the IVC stent both maintained patency and avoided ascites recurrence. In group B, six cases of ascites disappeared in the first week; another 15 cases did not disappear until after IVC stent deployment. After patients of all the two groups completed with DIPS plus IVC stenting, there was no comparison with the HE and the survival rates

### 3.1. Technical Success

**Figure 1:**
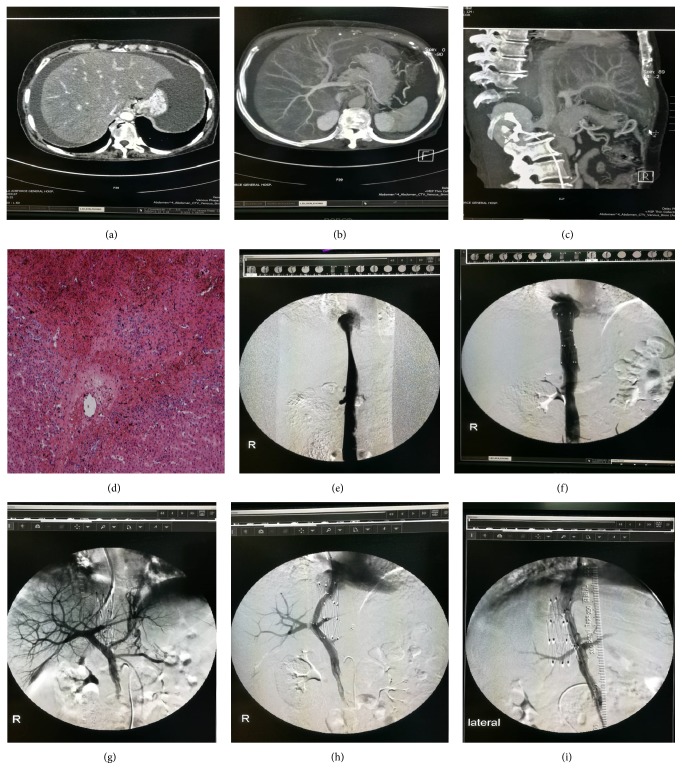
**DIPS placement combined with IVC stenting in SOS.** A female patient, aged 44 years, presented with botanical hepatotoxicity of pyrrolizidine-alkaloid-related decompensated cirrhosis. (a–c) CT shows liver enlargement, thinning portal vein, narrowed hepatic segment of IVC, presence of ascites, outflow obstruction of hepatic vein, and patchy signal enhancement in the absence of hepatic vein occlusion. (d) Specimen obtained from percutaneous transhepatic biopsy before treatment. High power image (100×, hematoxylin–eosin staining) showing dilatation of sinusoids and necrosis of hepatocytes (long arrow). Terminal hepatic vein was occluded (short arrow), but collagen deposition had not yet occurred. (e) IVC stenosis (black arrow). (f, g) After stent implantation of IVC, the intrahepatic left portal vein was punctured through the IVC stent. (h,i) DIPS stent implantation (black arrow indicates spring coil in the left gastric vein after embolization; venography shows that the variceal collateral vessel was not manifested).

The technical success rate in all 89 patients was 100% and there were no technical failures or complications. There were no deaths during the perioperative period. No patient died within 30 days after DIPS and IVC stenting, with an early survival rate of 100% (Figure 1). There was no IVC stent migration, despite five cases of IVC stent deformation during follow-up, but it did not affect IVC blood flow and DIPS stenting.

### 3.2. Changes of IVC Pressure and PSG

In group A, the mean pressure of the distal IVC before IVC stent deployment was 17.07±3.93 mmHg and then dropped to 3.83±3.71 mmHg after stent placement (*P*⩽0.001). In group B, 21 patients underwent DIPS placement without IVC stenting. Mean pressure of the distal IVC before and after DIPS deployment was 15.31±3.13 and 16.41±6.10 mmHg, respectively, but this difference was not significant (*P*=0.248). After IVC stent placement, the mean pressure of the distal IVC dropped to 4.15±3.79 mmHg, and there were significant differences before and after IVC stent placement (*P*=0.001). The mean interval times were 6 months between DIPS and IVC stenting. Mean PSG decreased from 44.97±11.87 to 24.88±5.28 mmHg and 45.88±9.05 to 24.13±3.91 mmHg in group A (*P*⩽0.001) and group B (*P*=0.001), respectively. Compared with two groups after IVC stent placement, there were no significant changes in mean pressure of the distal IVC (*P*=0.118) or PSG (*P*=0.604) (Table 2).

### 3.3. Improvement of Liver Function

In group A, mean AST, ALT, and TB concentration dropped from 383.98±289.49 to 249.73±192.12 U/L, 25.44±10.99 to 28.61±9.03 U/L, and 20.46±8.44 to 3.23±1.53 *μ*mol/L, respectively (*P*=0.001,* P*=0.003, and* P*=0.005). In group B, mean concentration of AST, ALT, and TB concentration dropped from 301.05±214.01 to 182.05±81.30 U/L, 26.31±10.91 to 20.00±9.40 U/L, and 20.33±8.01 to 3.40±1.59 *μ*mol/L (*P*=0.001,* P*=0.002, and* P*=0.004) at 14 days after shunt treatment, respectively (*P* < 0.05). There were no significant changes in AST, ALT, and TB level between the two groups (*P*=0.595,* P*=0.159 and* P*=0.192) (Table 3).

### 3.4. Improvement of Symptoms

In group A, ascites disappeared in the first week without paracentesis. In group B, six (28.57) cases of ascites disappeared in the first week; another 15 cases ascites did not disappear until after IVC stent deployment. There was a significant difference between the two groups of ascites disappear (*P*=0.004). The recurrence rate for bleeding was 10.29% in group A compared to 9.52% in group B (*P*=0.651), and the recurrence rate for ascites was 16.18% in group A compared to 14.29% in group B (*P*=0.616) (Table 3).

### 3.5. Shunt and Stent Dysfunction

**Figure 2:**
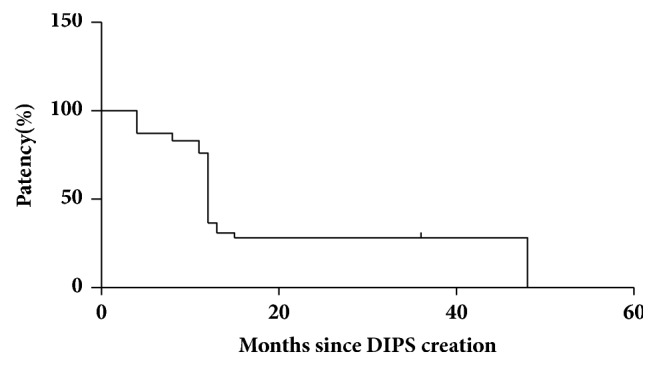
**Primary patency of DIPS stent and survival time of patients with SOS.** Mean DIPS stent primary patency was 21.028 months; the median DIPS stent primary patency was 12 months (95% confidence interval 17.01–25.04).

Total shunt malfunction occurred in 38 of 89 patients (53.52%) and the mean DIPS stent primary patency was 21.028 months (95% confidence interval=17.01–−25.04) (Figure 2). Two cases had stenosis at both ends of the stent in the hepatic tract, 19 had proximal end stenosis, 11 had distal end stenosis, and six had DIPS stent occlusion. One patient had IVC stent dysfunction. Patients with DIPS stent dysfunction were treated with balloon dilation (n=21) and stent replacement in the hepatic tract (n=19). The patient with IVC stent dysfunction underwent balloon dilation, and, after stent revision, their symptoms disappeared.

### 3.6. HE

HE occurred in 18 patients in group A and four in group B during follow-up. The incidence of HE did not differ significantly between group A and group B (20.59% versus 19.48%) (*P*=0.848) (Table 4). After drug treatment, the symptoms disappeared in patients with grade I or II HE. In patients with grade III HE, after two stents of shunt reduction were implanted, the symptoms disappeared.

### 3.7. Overall Survival

**Figure 3:**
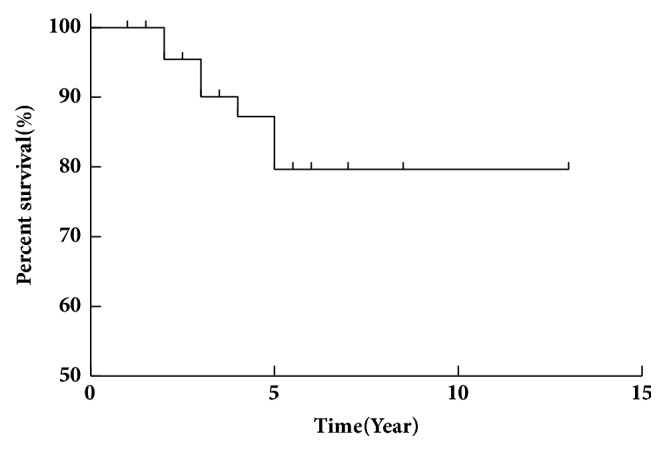
**The total survival time of cases with SOS.** The 1-year survival rate was 98%, 3-year survival rate was 89.59%, and 5-year survival rate was 80%.

One patient (1.41%) was lost to follow-up. One patient died within 1 year. Since the endpoint of this study, nine patients (12.68) have died. The longest follow-up was 13 years. The 1-year survival rate was 98%, 3-year survival rate was 89.59%, and 5-year survival rate was 80% (Figure 3).

## 4. Discussion

Our results provide evidence that DIPS combined with IVC stenting can be performed safely and achieve good clinical results and survival rates in patients with SOS-related to botanical hepatotoxicity. This is not consistent with the results of some previous studies [13], although previous studies about TIPS in SOS associated with herbal hepatotoxicity have been rare. Nevertheless, this does not mean that TIPS has no clinical benefit for SOS. These studies have focused more on patients treated with HSCT, bone marrow transplantation, radiation-induced liver disease, chemotherapy, or after solid organ transplantation.

It has been shown that TIPS might be considered as part of a protocol in patients with SOS and multiorgan failure who have no hope of survival [14]. The effect of SOS and multiorgan failure on survival, however, is limited since most patients die from extrahepatic causes [15]. In view of this, recent reviews of clinical practice guidelines [16, 17] have not recommended TIPS for SOS. This is because TIPS has not been shown to change the prognosis in patients with SOS. Nevertheless, SOS can be seen in other settings, in which TIPS may offer potentially useful treatment. The etiology of SOS caused by herbal hepatotoxicity, for example,* Gynura segetum* [18], is not the same as that caused by HSCT, bone marrow transplantation, radiation-induced liver disease, chemotherapy, or after solid organ transplantation. If the evolution of the disease is not the same, then neither is the clinical course. SOS induced by herbal hepatotoxicity can be divided into acute, subacute, and chronic stages. Mild cases of SOS associated with herbal hepatotoxicity can be cured by medical treatment [19], and, for serious cases of SOS in patients who often manifest subacute or chronic diseases, interventional treatment can be chosen.

In the past, we have lacked awareness of SOS, since it is easy to confuse with Budd–Chiari syndrome (BCS), which has resulted in misdiagnosis. Since the clinical symptoms of SOS are easily confused with those of BCS [20], we used the DIPS procedure to treat SOS, which has also been used to treat BCS [21]. Clinical presentation of hepatotoxicity caused by herbal products is nonspecific and is similar to the presentation of BCS symptoms [22]. The interventional procedure is often similar between BCS and SOS, and both are treated through the strut of the preexisting IVC stent. After IVC stenting, the symptoms were relieved. If there was IVC stenosis or occlusion, the IVC was recanalized, and, after 1 week, the DIPS procedure was performed. Such a process assumed that the IVC stent was stable and did not migrate, guaranteeing the safety of the DIPS procedure. During DIPS and follow-up, there was no case of IVC stent migration, despite five cases of IVC stent deformation.

In the traditional TIPS procedure, the puncture needle starts from the right hepatic vein and proceeds to the right branch of the portal vein, and then the stent is placed [23]. A major advantage of DIPS is that it does not involve the hepatic vein, so the technique is especially useful in hepatic vein obstruction. The improvement of the creation of a side-to-side shunt between the IVC and left branch of the portal vein was intended to reduce the incidence of HE and increase TIPS stent-free patency [24, 25]. Also, the DIPS shunt is short, smooth, and straight, which reduces stenosis and occlusion of the shunt due to shear stress. If DIPS is placed first, followed by IVC stenting, the latter may affect the stability of both stents, thereby affecting IVC blood flow.

We showed that mean AST, ALT, and TB concentrations dropped at 14 days after shunt treatment, and ascites and variceal bleeding disappeared. This showed that IVC stenting combined with DIPS could have a good clinical effect for patients with SOS associated with botanical hepatotoxicity, which confirms the results of some previous studies of TIPS [26, 27]. In the present study, the levels of AST and ALT were higher than for other causes of liver cirrhosis, but TB was not higher. The cause of this phenomenon warrants further study. In our study, HE was still a problem despite its low incidence compared with other diseases that TIPS cannot completely cure [28].

With regard to survival, our results were inconsistent with those of previous studies [1, 10, 12, 14]. Because all the cases in our study demonstrated subacute or chronic symptoms, there were no early-stage patients included. Compared with a recent systematic review of Tusanqi-related SOS [29], we found that cumulative survival rate was higher. This may be because all the patients in the previous study received medical treatment, and there was no staging of the disease, or some cases were not treated with TIPS or DIPS. The interval between the onset of SOS and DIPS placement has not been mentioned, and only one small report [30] has specifically commented on this. Some have recommended [14, 31] that SOS should be treated with TIPS as early as possible. The interval could influence the survival rate [32], and if multiorgan failure is already present, then patients are probably being treated too late [33]. In our study, because there was no patient with acute lesions, the relevant information and DIPS treatment were not available, which should be the goal of our next study.

Pyrrolizidine alkaloids are present in distinct plant families that grow worldwide [34], and although they are sporadic in western countries, pyrrolizidine alkaloid poisoning attracts sufficient attention [35, 36]. Whether SOS is caused by herbal medicines containing pyrrolizidine alkaloids or other factors [4, 5], its pathological process is similar and leads to elevated portal pressure [37]. DIPS improved the symptoms of SOS in our study and resulted in better prognosis; however, these results were inconsistent with some previous studies [13]. We believe that in the Far East or western countries, regardless of the cause of SOS, the results of our study will be useful for elimination of extrahepatic complications.

This study had several limitations. First, randomized controlled trials are needed to verify our results. Second, early SOS-related decompensated cirrhosis due to botanical hepatotoxicity was not studied in patients treated with combined IVC stenting and DIPS. Finally, clinical characteristics and outcomes were not compared between patients with herbal hepatotoxicity-related SOS and those with SOS with other etiology.

In conclusion, in patients with SOS-related decompensated cirrhosis of botanical hepatotoxicity, the use of IVC stenting combined with DIPS placement was associated with significant reduction of symptoms.

## Figures and Tables

**Table 1:** Baseline characteristics of SOS cases.

Characteristic/group	A	B	*P* value
Gender M/F	30/38	9/12	0.366
Age (mean (S.D.))	38.98±19.34	35.25±8.64	0.299
Child-PughA/B/C	3/55/10	1/11/9	0.328
MELD score (mean (S.D.))	12.49±7.25	13.17±9.16	0.145
Accompanied viral hepatitis	5	3	0.628
Chronic ethanol consumption	6	0	0
Cryptogenic hepatitis	2	0	0
Variceal hemorrhage(VH)	30	5	0.217
Refractory ascites(RA)	65	14	0.134
Both VH and RA	25	2	0
Laboratory tests			
Alanine transaminase	249.73±192.12	182.05±81.30	0.595
Aspartate transaminase	383.98±289.49	301.05±214.01	0.604
Alkaline phosphatase	258.32±163.24	264.43±176.91	0.371
Gamma glutamyl transpeptidase	276.45±125.34	297.39±136.73	0.256
Total bilirubin	25.44±10.99	26.31±10.91	0.192
Albumin	29.37±9.25	28.61±9.72	0.261
Prothrombin time	16.24±7.69	18.15±8.46	0.135
Clinical presentations			
Abdominal distention	67	18	0.154
Abdominal pain	24	13	0.364
Weakness	51	16	0.412
Poor appetite	63	19	0.346
Jaundice	48	12	0.216
Hepatomegaly	65	18	0.423
Splenomegaly	31	13	0.338
Pleural effusion	2	5	0
Lower limbs edema	12	3	0.026
Endoscopic therapy	48	15	0.128
Asites paracentesis	154	34	0.093
Seattle criteria	24	13	0.364
Baltimore criteria	18	9	0.379

No difference (P>0.05) could be seen in terms of age, sex, Child–Pugh score, and MELD score; laboratory tests, and clinical presentations. MELD, Model of End-stage Liver Disease; RA, refractory ascites; VH, variceal hemorrhage; laboratory tests and clinical presentations.

**Table 2:** Treatment outcomes in two groups.

Outcomes/groups	A		*P* value	B		*P* value
	Before	After		Before	After	
				15.31±3.13	16.41±6.10	0.248
IVC (mmHg)	17.07±3.93	3.83±3.71	≤0.001	16.41±3.79	4.15±3.79	0.001(0.018)
PSG (mmHg)	44.97±11.87	24.88±5.28	≤0.001	45.88±9.05	24.13±3.91	0.001(0.604)

**Table 3:** Treatment outcomes in two groups.

Outcomes/groups	A		*P* value	B		*P* value
	Before	After		Before	After	
AST( U/L)	383.98±289.49	28.61±9.03	0.001	301.05±214.01	20.00±9.40	0.001(0.604)
ALT (U/L)	249.73±192.12	20.46±8.44	0.003	182.05±81.30	20.33±8.01	0.002(0.595)
TB (*μ*mol/L)	25.44±10.99	3.23±1.53	0.005	26.31±10.91	3.40±1.59	0.004(0.192)

**Table 4:** Outcomes of symptoms in two groups.

Symptoms/group	A	B	*P* value
Absorption of ascites	68(100%)	6(28.57%)	0.004
Recurrence rate for bleeding	7(10.29%)	2(9.52%)	0.651
Recurrence rate for ascites	11(16.18%)	3(14.29%)	0.616
HE	14(20.59%)	4(19.48%)	0.848
